# Sleep dimensions are associated with obesity, poor diet quality and eating behaviors in school-aged children

**DOI:** 10.3389/fnut.2022.959503

**Published:** 2022-09-23

**Authors:** Catalina Ramírez-Contreras, Alicia Santamaría-Orleans, Maria Izquierdo-Pulido, María Fernanda Zerón-Rugerio

**Affiliations:** ^1^Department of Nutrition, Food Science and Gastronomy, Food Science Torribera Campus, University of Barcelona, Barcelona, Spain; ^2^INSA-UB, Nutrition and Food Safety Research Institute, University of Barcelona, Barcelona, Spain; ^3^Laboratorios Ordesa, Scientific Communication Department, Barcelona, Spain

**Keywords:** sleep disturbances, sleep duration, sleep pattern, body mass index, eating behaviors, diet quality, children

## Abstract

**Objectives:**

The aim of this cross-sectional study was to investigate the association between sleep dimensions (duration, patterns, and disturbances) with body mass index (BMI), diet quality, and eating behaviors in school-aged children. Additionally, we aimed to investigate whether obesogenic eating behaviors (higher food responsiveness, lower satiety responsiveness, and less slowness in eating) and poor diet quality could mediate the potential association between sleep and obesity in school-aged children.

**Materials and methods:**

For all participants (*n* = 588 children, age 5–12 years; 51% girls) we evaluated: sleep dimensions, BMI, diet quality, eating behaviors (food responsiveness, satiety responsiveness and slowness in eating). Linear regression models were used to test associations between exposure and outcome variables. Additionally, path analysis was conducted to test whether eating behaviors mediated the relationship between sleep and obesity.

**Results:**

Shorter sleep duration (β = −0.722, *p* = 0.009) and greater sleep disturbances (β = 0.031, *p* = 0.012) were significantly associated with BMI. Additionally, we observed that diet quality was significantly associated with sleep duration (β = 0.430, *p* = 0.004), the midpoint of sleep (β = −0.927, *p* < 0.001), and sleep disturbances (β = −0.029, *p* < 0.001). Among other findings, greater sleep disturbances were associated with food responsiveness (β = 0.017, *p* < 0.001), satiety responsiveness (β = 0.015, *p* < 0.001), and slowness in eating (β = 0.012, *p* < 0.001). Importantly, food responsiveness was found as significant mediator of the relationship between sleep and BMI (*R* = 0.427, *R*^2^ = 0.182, *p* < 0.001).

**Conclusions:**

Late sleep patterns, short sleep duration, and greater sleep disturbances are significantly related with *what* and *how* school-aged children eat. Importantly, poor diet quality was significantly related to all three sleep dimensions, while eating behaviors had a significant relationship with greater sleep disturbances. These findings may be relevant to the development of behavioral targets to prevent childhood obesity, including sleep hygiene guidelines as a strategy to improve children's eating habits, as well as their BMI.

## Introduction

Sleep is an essential component of healthy development and the general wellbeing of children (1). Additionally, sleep is important for children's learning, memory process, and school performance (2). In children, sleep is considered adequate when the recommended duration of sleep (9–11 h per day) is met (3). Furthermore, considering that in today's society facilities are available around the clock and lifestyle demands (e.g., school start times and extracurricular activities) may delay sleep/wake patterns (4–8), beyond sleep duration, other dimensions such as sleep patterns and disturbances, have become equally important to consider (9). On the one hand, sleep patterns refer to sleep/wake schedules, while sleep disturbances refer to different factors including sleep fragmentation (i.e., arousals or awakenings), sleep disorders (e.g., sleep apnea and parasomnias), and poor sleep quality (9). Unfortunately, the prevalence of sleep disturbances in school-aged children is about ~37%, with bedtime resistance, sleep-onset delay, and daytime sleepiness being the most common sleep problems in this stage of life (2). Understanding the implications of sleep disturbances in childhood is essential, especially considering that the consequences include: headaches, behavioral problems, and poor academic performance (1, 2). Furthermore, during early adolescence (9–12 years old) insufficient sleep could have adverse effects on neurocognitive development in children (10).

Furthermore, it is well-known that inadequate sleep can negatively affect eating habits (11–15). Not surprisingly, short sleep duration, late sleep patterns, and the presence of sleep disturbances is significantly related with higher body mass index (BMI) in children (9, 14, 16–19). Among a wide range of mechanisms underlying the association between inadequate sleep and obesity, the increased hedonic drive for food may explain why people who have not slept well-tend to have a poor diet quality (20, 21). Along these lines, St-Onge et al. (22) pointed out that inadequate sleep alters neuronal activity, which predisposes individuals to enhanced susceptibility to food stimuli. Additionally, recent evidence has shed light on eating behaviors as potential mediators of the relationship between sleep and obesity in adults (23–25). Accordingly, poor sleep quality is significantly related with obesogenic eating behaviors (such as emotional eating and disinhibited eating behaviors), which in turn, could lead to obesity (26, 27). In children, obesogenic eating behaviors such as external eating and food responsiveness have been associated with poor sleep outcomes (21, 24). However, it has yet to be elucidated whether these behaviors could also be the missing link in the relationship between sleep and obesity in childhood. Note that both behaviors describe the child's preference for tasty foods and the tendency to eat when prompted by external cues (28). Consequently, these obesogenic eating behaviors are associated with higher BMI (25, 28–30).

Interestingly, children's eating behavior is also characterized by other food avoidant behaviors such as satiety responsiveness and slowness in eating, both of which have been associated with lower BMI (28–30). However, unlike food responsiveness, the association between these food avoidant eating behaviors and sleep is inconsistent (23, 25, 31). Two studies in children aged 2 and 5 years (23, 25), found no significant association between sleep duration and satiety responsiveness and slowness in eating. While, on the other hand, one study pointed out that well-rested children with obesity were better able to avoid unnecessary food intake (31). Thus, more evidence needs to be provided regarding the association between food avoidant behaviors and sleep.

Therefore, the aim of our study was to investigate the association of sleep dimensions (duration, patterns, and disturbances) with BMI, diet quality, and eating behaviors in school-aged children. We hypothesized that shorter sleep duration, later sleep patterns, and more sleep disturbances would be associated with higher BMI, poor diet quality, and obesogenic eating behaviors (greater food responsiveness, less satiety responsiveness, and less slowness in eating). In addition, we aimed to investigate whether obesogenic eating behaviors and poor diet quality could mediate the potential association between sleep and obesity in school-age children.

## Materials and methods

### Participants and study design

This is a cross-sectional study, where participants were recruited by convenience sampling, to complete a web-based questionnaire. The link to the survey was shared during the academic year (between May 2021 and June 2021) with the parents/caregivers of school-aged children (5–12 years old) who were subscribed to Laboratorios Ordesa Family Club. The latter is a web platform addressed to pregnant women, parents and/or caregivers of infants and children, which is intended to promote healthy eating. The parents or caregivers of eligible children received an e-mail with a brief explanation of the project to encourage them to participate, as well as the link to answer the questionnaire. E-mails were sent progressively over a 1 week period in order to avoid receiving too many answers at one time. Also, note that at the time that we collected the data, the schools were in person.

The inclusion criteria consisted of being a parent of a child between 5 and 12 years of age and being willing to participate in the study. Based on these criteria, a total of 687 school-aged children whose caregivers provided informed consent and the information required for the development of the study were eligible for this study. Upon data inspection, a total of 99 participants were excluded (78 who were out of the age range and 26 who provided incorrect information regarding weight and height), resulting in a final analytical sample of 588 participants.

### Ethical aspects

Participation in the study was entirely voluntary and anonymous. In addition, all the study procedures were conducted according to the general recommendations of the Declaration of Helsinki and were approved by Ethics Committee of the University of Barcelona (IRB00003099). Written informed consent to participate in this study was provided by the participants' legal guardian/next of kin.

### Data collection

We used Open Data Kit (ODK) (32), an open-source software, to develop an online screening tool that included questions on child weight, height, age, gender, and sleep variables. In addition, we included a series of validated questionnaires (detailed below) to assess sleep disturbances, diet quality, eating behaviors, and physical activity. ODK has a user-friendly web interface for designing web forms and programming simple logic.

### Outcome variables

#### Body mass index

Weight and height were asked in a questionnaire as follows: “What is your child current weight? (in kg)” and “What is your child current height? (in cm).” Height and weight were used to calculate the BMI (kg/m^2^) as follows: weight (kg) divided by height squared (m^2^). Children's BMI was then classified according to the International Obesity Taskforce into: “underweight,” “normal weight,” “overweight,” and “obesity” according to specific age and gender BMI cut-off criteria (33).

#### Diet quality

Diet quality was evaluated through the Mediterranean Diet Quality Index in children and adolescents (KIDMED) (34). This test is based on the principles that sustain Mediterranean dietary pattern and those that undermine them. This questionnaire consists of 16 items which are answered as “Yes” or “No” questions. Subsequently, items denoting less adherence are assigned a value of−1, while those related to greater adherence are scored +1. The total score ranges from −4 to 12, where higher scores indicate greater adherence to the Mediterranean Diet. In addition, adherence to the Mediterranean Diet was classified according to the score as follows: “poor” (≤3 points), “average” (4–7 points), or “good” (≥8 points).

#### Eating behaviors

Eating behaviors were evaluated through the shortened Spanish version of the Children's Eating Behavior Questionnaire (CEBQ) (28). This version of the CEBQ contains 14 questions (detailed in Supplementary Table 1) which are rated on a five-point Likert scale ranging from 1 (“never”) to 5 (“always”). The latter are used to evaluate the following subscales:

- Food responsiveness: which evaluates general appetite levels that might be considered as maladaptive in children, such as preference for palatable (tasty) foods and a tendency to eat when prompted by external cues. This subscale was evaluated through 5 items.
- Satiety responsiveness: which assesses the degree to which children respond to physiological cues of fullness or choose to stop eating based on perceived fullness. This subscale was evaluated through 5 items.
- Slowness in eating: which evaluates the tendency to eat more slowly during a meal and prolong meal duration, indicating a lack of interest in eating. This subscale was evaluated through 4 items.

Note that scores were calculated separately for each subscale as a mean of all items, where higher scores represented a higher expression of that behavior.

#### Physical activity

Physical activity was assessed with the Physical Activity Unit 7 Item Screener (PAU−7S), which has been validated in Spanish children and adolescents (35). The questions refer to the usual opportunities to be physically active during the day, including individual and group activities. Moderate to Vigorous Physical Activity (MVPA min/day) was calculated based on the sum of all activities except walking.

### Exposure variables

#### Sleep patterns

We used the midpoint of sleep (local time) as a marker of sleep patterns. Parents reported bedtime and wakeup time in weekends and weekdays. Then, we calculated each participant's midpoint between bedtime and wake up time during weekdays and weekends. A total weekly midpoint of sleep was calculated as: [5 × weekday midpoint of sleep (h) + 2 × weekend midpoint of sleep (h)]/7. In this case, the later the midpoint of sleep, the later the bed and wakeup timing.

#### Sleep duration

Parent-reported sleep duration was defined as the difference between bedtime and wakeup time on weekdays/weekends in hours. The average sleep duration was calculated as follows: [5 × weekday sleep (h) + 2 × weekend sleep (h)]/7 (36). In this case, lower values indicated shorter sleep duration. Additionally, short sleep duration was considered if the child slept < 9 hours per day (3).

#### Sleep disturbances

The Sleep Disturbances Scale for Children (SDSC) is a well-validated parental report instrument to assess children's sleep disturbances (37). This questionnaire contains 26 items which are rated on a Likert scale ranging from 1 (“never”) to 5 (“always”). Subsequently, answers are grouped into six factors: disorders of initiating and maintaining sleep; sleep breathing disorders; arousal disorders; sleep-wake transition disorders; disorders of excessive somnolence; sleep hyperhidrosis. The definitions of sleep factors are provided in Supplementary Table 2. Total SDSC score ranged from 26 to 130, where higher values reflect higher frequency of sleep disturbances. Additionally, total SDSC scores > 39 are indicative of sleep disturbances.

### Statistical analysis

Normality was confirmed for all variables by histograms and Q-Q plots. Descriptive characteristics are presented for all participants, including mean and standard deviation for continuous variables and proportions for categorical variables. First, we tested associations between outcome and exposure variables using linear regression models. Then, partial correlations were used to test associations between the six SDSC sleep factors with BMI and eating behaviors. Furthermore, we used general linear models to test associations between sleep dimensions and the KIDMED items. We then corrected *p*-values for multiple comparisons using the Benjamini–Hochberg method, assuming a False Discovery Rate (FDR) of 5%.

Subsequently, we tested whether those variables that were significantly associated with the BMI (food responsiveness, satiety responsiveness, and slowness in eating) were significant mediators of the association between sleep disturbances and BMI (Supplementary Figure 1). Full mediation was claimed if (i) the exposure was correlated with the outcome; (ii) the exposure was correlated with the mediator; (iii) the mediator was correlated with the outcome; and (iv) the association of the exposure with the outcome adjusting for the mediator was sufficiently close to zero (the mediator mediates the exposure-outcome relationship). Analyses were conducted separately for each mediator, using the PROCESS macro (38) version 3.3 for SPSS. All analyses were adjusted for gender, age, and physical activity. All analyses were performed using SPSS statistical computer software, version 25.0 (IBM SPSS Statistics, Armonk, NY, USA). The significance testing was considered when *p* < 0.05.

## Results

A total of 588 school-aged children (7.5 ± 2.1 years) were included in this cross-sectional study, 51% of whom were girls (Table 1). Overall, 56% of the participants had normal weight, while 15.2% had underweight, and the remaining 28.8% had overweight or obesity. Bedtime on weekdays was at 21:58 ± 00:52 and on weekends at 22:46 ± 01:00. Meanwhile, wakeup time on weekdays was at 07:50 ± 00:38 and on weekends at 09:00 ± 01:07. Regarding sleep dimensions (Table 1), the midpoint of sleep was at 03:09 ± 00:29 and average sleep duration was 10.0 ± 0.6 h/day, with 94.7% of children meeting the age-appropriate sleep recommendations. Concerning sleep disturbances, the mean SDSC total score was 41.1 ± 10.9 points. Noteworthy, almost half of the children (49.3%) presented sleep disturbances, being the disorders of initiating and maintaining sleep and sleep-wake transition disorders the sleep factors that presented the highest scores (10.8 ± 3.4 points and 11.2 ± 4.2 points, respectively), followed by the disorders of excessive somnolence (7.2 ± 2.7 points) (Table 1).

**Table 1:** Characteristics of the population studied.

**Total sample, n**	**588**
Age, years	7.5 (2.1)
Gender, % girls	51.0
Body mass index, kg/m^2^	17.1 (3.4)
Sleep dimensions	
Sleep patterns, midpoint of sleep (hh:mm)	03:09 (00:29)
Sleep duration, h	10.0 (0.6)
Sleep disturbances, total SDSC score	41.1 (10.9)
Disorders of initiating and maintaining sleep, score	10.8 (3.4)
Sleep breathing disorders, score	4.2 (1.7)
Arousal disorders, score	3.9 (1.3)
Sleep-wake transition disorders, score	11.2 (4.2)
Disorders of excessive somnolence, score	7.2 (2.7)
Sleep hyperhidrosis, score	3.9 (2.2)
Diet quality, score	7.5 (2.2)
Eating behaviors	
Food responsiveness, score	2.4 (0.9)
Satiety responsiveness, score	2.5 (0.7)
Slowness in eating, score	2.8 (0.9)
Physical activity, MVPA min/day	143.4 (61.1)

BMI, Body mass index; SDSC, Sleep Disturbance Scale for Children; MVPA, moderate to vigorous physical.

Regarding diet quality, half of the children (50.5%) had a good adherence to the Mediterranean diet, followed by 44.7% who had an average adherence, and only 4.8% had poor adherence to this dietary pattern. Table 1 also shows the mean scores for food responsiveness, satiety responsiveness and slowness in eating. In terms of physical activity, we observed that children engaged in moderate to vigorous physical activity for an average of 143.4 min/day.

### Higher BMI is significantly associated with short sleep duration and greater sleep disturbances

As shown in Table 2, the sleep dimensions that were significantly associated with BMI were sleep duration and sleep disturbances. Consequently, 1-h decrease in sleep duration was associated with higher BMI (β = −0.722 [95% CI: −1.158, −0.286], *p* = 0.009), while a 1-point increment in SDSC score was associated with higher BMI (β = 0.031 [95% CI: 0.007, 0.056], *p* = 0.012). Interestingly, partial correlation analyses revealed that among the sleep factors that characterize the SDSC questionnaire, disorders of initiating and maintaining sleep (*r* = 0.102, *p* = 0.024) and sleep breathing disorders (*r* = 0.096, *p* = 0.032) were positively related to BMI (Supplementary Table 3).

**Table 2:** Associations between sleep dimensions and body mass index, eating behaviors, and diet quality in school-aged children.

	**Midpoint of sleep, hh: mm**	**Sleep duration, h**	**Sleep disturbances, SDSC score**
	**β [95% CI]**	**β [95% CI]**	**β [95% CI]**
BMI, kg/m^2^	0.520 [−0.034, 1.073]	−0.722 [−1.158, −0.286]**	0.031 [0.007, 0.056]*
Eating behaviors
Food responsiveness, score	−0.112 [−0.267, 0.044]	−0.146 [−0.270, −0.022]	0.017 [0.011, 0.024]***
Satiety responsiveness, score	0.106 [−0.016, 0.227]	−0.070 [−0.167, 0.028]	0.015 [0.010, 0.021]***
Slowness in eating, score	0.025 [−0.121, 0.171]	−0.038 [−0.154, 0.079]	0.012 [0.005, 0.018]***
Diet quality, KIDMED score	−0.927 [−1.285, −0.569]***	0.430 [0.140, 0.720]**	−0.029 [−0.045, −0.012]***

BMI, Body mass index; CI, confidence interval; SDSC, Sleep Disturbance Scale for Children. Data was analyzed using linear regression models to test associations between sleep dimensions, BMI, eating behaviors, and diet quality. Analyses were adjusted for age, gender, and physical activity. The table shows the unstandardized coefficient (β), 95% CI, and p-value associated with each predictor variable.

^*^p < 0.05,

^**^p < 0.01,

^***^p < 0.001.

### Eating behaviors are associated with greater sleep disturbances

Among other findings (Table 2), we observed that sleep disturbances were the only sleep dimension that was significantly related to eating behaviors. More specifically, our results revealed that 1-point increment in SDSC score was positively associated with food responsiveness (β = 0.017 [95% CI: 0.011, 0.024], *p* < 0.001), satiety responsiveness (β = 0.015 [95% CI: 0.010, 0.021], *p* < 0.001), and slowness in eating (β = 0.012 [95% CI: 0.005, 0.018], *p* < 0.001). Interestingly, partial correlation analyses showed that, among the sleep factors that characterize SDSC questionnaire, disorders of initiating and maintaining sleep and sleep-wake transition disorders were significantly associated with food responsiveness (*r* = 0.160, *p* < 0.001 and *r* = 0.164, *p* < 0.001, respectively), satiety responsiveness (*r* = 0.225, *p* < 0.001 and *r* = 0.169, *p* < 0.001, respectively), and slowness in eating (*r* = 0.112, *p* = 0.015 and *r* = 0.107, *p* < 0.001). Furthermore, we found that disorders of excessive somnolence were significantly associated with both satiety responsiveness (*r* = 0.187, *p* < 0.001) and slowness in eating (*r* = 0.157, *p* < 0.001).

### Poor diet quality is associated with late sleep patterns, short sleep duration, and greater sleep disturbances

**Figure 1:**
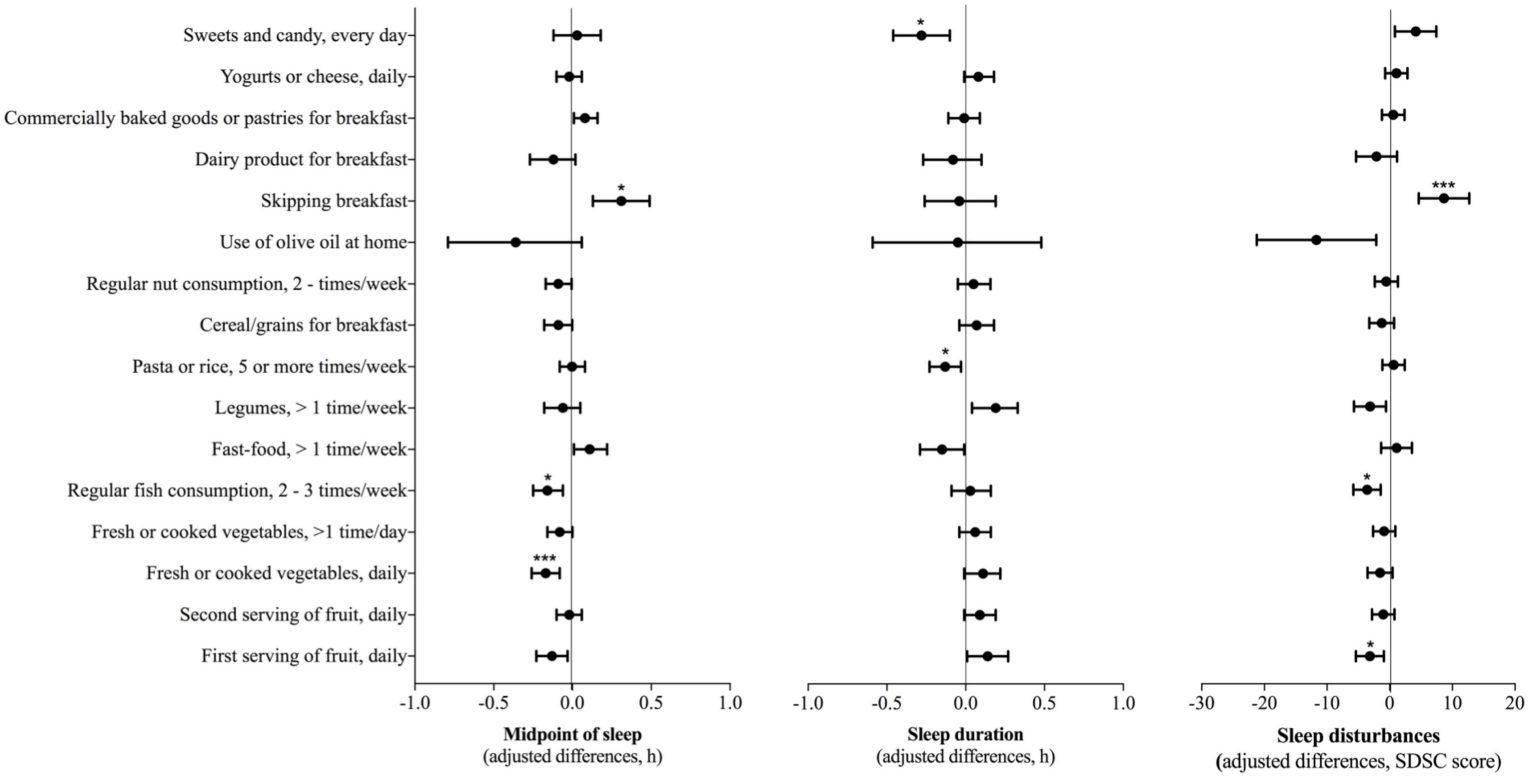
Associations between sleep dimensions and the 16 items of the Mediterranean Diet Quality Index. General linear models adjusted for age, gender, and physical activity were conducted to test these associations. The figure shows the unstandardized coefficient (β), 95% CI, and adjusted *p*-values associated with each predictor variable. *P*-values were corrected using the Benjamini–Hochberg method, assuming a False Discovery Rate (FDR) of 5%. *p < 0.05, ***p < 0.001.

We observed that the midpoint of sleep, sleep duration, and total SDSC score were significantly associated with diet quality (KIDMED score) (Table 2). A subsequent analysis showed significant associations between breakfast skipping and the delay in the midpoint of sleep (β = 0.310 [95% CI: 0.130, 0.49], *p* = 0.010), while regular fish consumption (β = −0.160 [95% CI: −0.250, −0.060], *p* = 0.016) and the daily consumption of fresh or cooked vegetables (β = −0.170 [95% CI: −0.260, −0.080], *p* < 0.001) were associated with the advance of the midpoint of sleep (Figure 1). Meanwhile, the daily consumption of sweets and candy (β = −0.280 [95% CI: −0.460, −0.100], *p* = 0.021) and having pasta or rice >5 times/week (β = −0.130 [95% CI: −0.230, −0.030], *p* = 0.048) were significantly associated with a decrease in sleep duration (Figure 1). Furthermore, regular fish consumption (β = −3.611 [95% CI: −5.787, −1.434], *p* = 0.010) and daily fruit consumption (β = −3.157 [95% CI: −0.913, −5.401], *p* = 0.036) were significantly associated with less sleep disturbances, breakfast skipping was significantly related with more sleep disturbances (β = 8.671 [95% CI: 4.643, 12.698], *p* < 0.001).

### Food responsiveness mediates the association between disturbances and higher BMI

**Figure 2:**
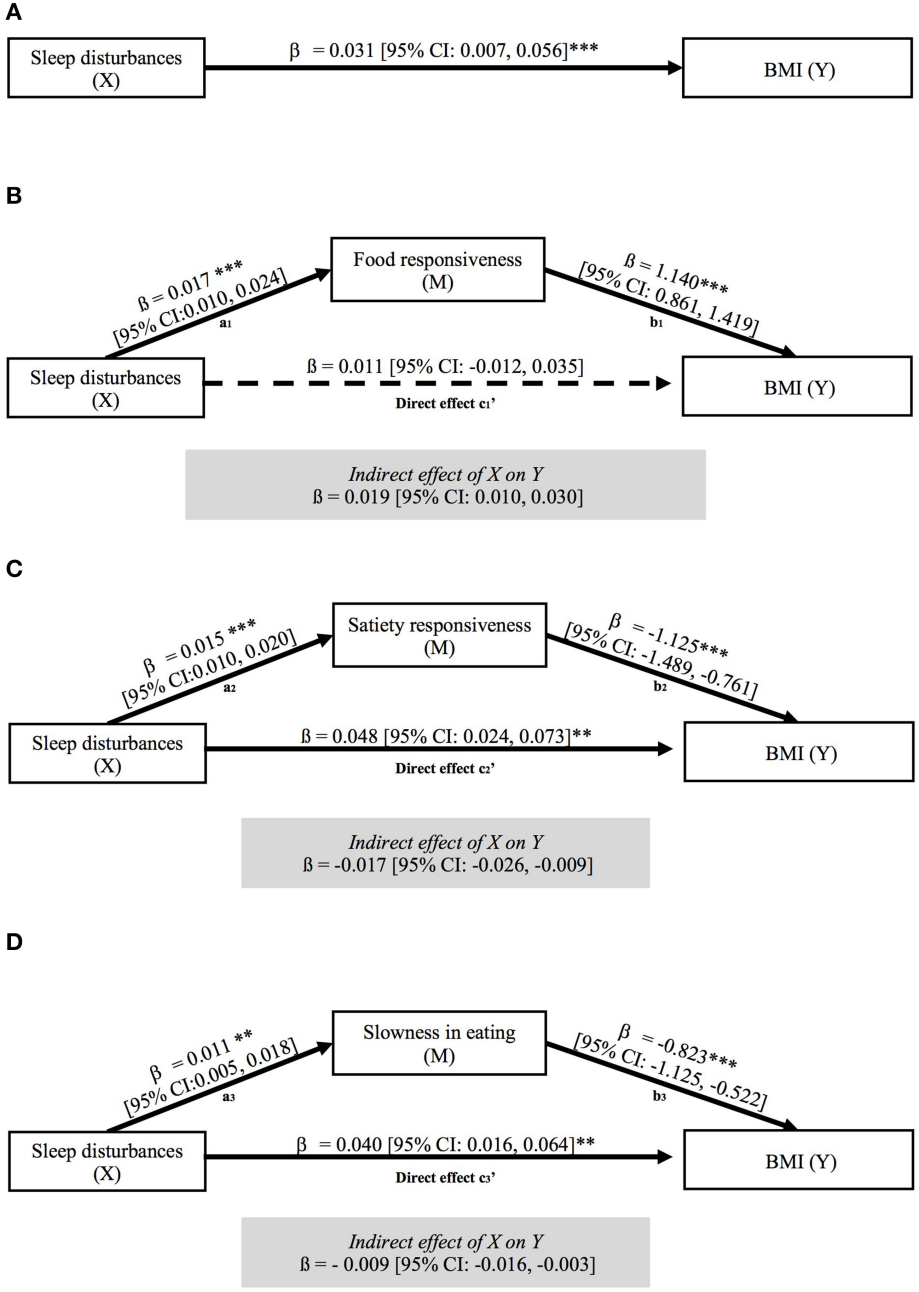
Mediation model highlighting the effect of sleep disturbances on BMI *via* food responsiveness, satiety responsiveness and slowness in eating. Figure (**A**) shows the direct effect of sleep disturbances on BMI, while the remaining figures show the effect of sleep disturbances on BMI via food responsiveness (**B**), satiety responsiveness (**C**) and slowness in eating (**D**). All models were adjusted for age, gender, and physical activity. Results are shown as unstandardized β coefficient represents with their 95% CI. Solid lines indicate statistically significant paths, while dotted lines indicate non-significant paths; ***p < 0.001; **p < 0.01, *p < 0.05.

As shown in Table 3, BMI was significantly associated with food responsiveness, satiety responsiveness, and slowness in eating, while no significant association was found between BMI and diet quality. Thus, we investigated whether eating behaviors mediated the association between sleep disturbances and BMI (Figure 2). The results revealed that only food responsiveness fully mediated the association between sleep disturbances and BMI (Figure 2B). As observed, paths a_1_ and b_1_ were statistically significant. Consequently, greater sleep disturbances were significantly associated with greater food responsiveness (ß = 0.017 [95% CI: 0.010, 0.024]). Meanwhile, greater food responsiveness was significantly associated with higher BMI (ß = 1.140 [95% CI: 0.861, 1.419]). Meanwhile, c_1_'-path was not statistically significant (Figure 2B) thus, there was a significant indirect association between poor sleep quality and higher BMI *via* food responsiveness (indirect effect = 0.02 [95% CI: 0.01, 0.03]). Note that ~18.2% of the variance in BMI was accounted by food responsiveness (*R* = 0.427, *R*^2^ = 0.182, *p* < 0.001).

**Table 3:** Associations between the body mass index and eating behaviors and diet quality in school-aged children.

	**BMI, kg/m** ^**2**^	
	**β [95% CI]**	***P*-value**
Eating behaviors
Food responsiveness, score	0.093 [0.071, 0.114]	< 0.001
Satiety responsiveness, score	−0.047 [−0.065, −0.030]	< 0.001
Slowness in eating, score	−0.053 [−0.074, −0.031]	< 0.001
Diet quality, score	−0.025 [−0.079, 0.029]	0.363

BMI, Body mass index; CI, confidence interval. Data was analyzed using linear regression models to test associations between BMI and eating behaviors and diet quality. Analyses were adjusted for age, gender and physical activity. The table shows the unstandardized coefficient (β), 95% CI and p-value associated with each predictor variable.

Regarding the mediation effect of satiety responsiveness (Figure 2C) and slowness in eating (Figure 2D), we observed that although paths a and b of the structural models were significant, so were c'_2_ and c'_3_ paths, therefore mediation could not be claimed.

## Discussion

The main contribution of this research work is that sleep dimensions: late sleep patterns, short sleep duration, and greater sleep disturbances are significantly related with *what* and *how* school-aged children eat. Importantly, poor diet quality was significantly related to all three sleep dimensions, while eating behaviors had a significant relationship with greater sleep disturbances. Furthermore, we showed that short sleep duration and more sleep disturbances were the two sleep dimensions that had a negative impact in children's BMI. Here, we also showed that food responsiveness is a significant mediator of the relationship between sleep disturbances and obesity in school-aged children. As such, children with greater sleep disturbances were more prone to eat palatable foods or respond to food cues (e.g., sight or smell) that, in turn, would lead them to obesity.

To our knowledge, this is the first study to show the mediating role of food responsiveness in the relationship between sleep and obesity in children. The latter would be consistent with Blumfield et al. (26) findings, who showed that disinhibited eating behavior mediated the association between poor sleep quality and obesity among adults. Accordingly, in the context of sleep impairment, an increased drive for exciting rewards in combination with disinhibited eating behavior can exacerbate food-seeking behavior, especially for palatable foods (26, 39). This would be also in line with previous research in toddlers (23) and young adults (27), which showed that poor sleep quality was associated with obesogenic eating behaviors, such as increased food responsiveness and greater uncontrolled eating. These findings also support recent evidence highlighting that inadequate sleep is associated with greater food intake *via* hedonic mechanisms, rather than homeostatic ones (20).

Equally interesting, eating behaviors related to a decrease in food consumption in response to satiety (known as “satiety responsiveness” and “slowness in eating”) were associated to greater sleep disturbances, although they did not play a role in the sleep-obesity relationship. This may be because these appetitive traits are associated with lower BMI (28–30), which is also consistent with our findings. Furthermore, Oberle et al. (31) noted that the response to these food avoidant behaviors was dependent of general fatigue symptoms and body fat percentages (31). The authors pointed out that children and adolescents with lower body fat percentage avoided more food when they were more fatigued, while those with a higher body fat percentage were able to avoid more food when they were less fatigued (31). According to our results, greater satiety responsiveness and slowness in eating were positively associated with excessive somnolence and the majority of our population (71%) was either underweight or normal weight. This could explain, in part, why in our study the sleep-obesity relationship was not mediated by satiety responsiveness and slowness in eating. However, we acknowledge that the relationship between sleep and eating behaviors is complex and thus, more evidence needs to be warranted.

Our results also revealed that more sleep disturbances and shorter sleep duration were significant determinants of poor diet quality among school-aged children, which is in agreement with other studies on the pediatric population (14, 27, 40, 41). Interestingly, we found that daily consumption of sweets and candy, as well as eating pasta or rice 5 or more times per week, were two dietary habits associated with shorter sleep duration. Evidence from experimental studies indicates that shorter sleep duration is associated with greater food attractiveness and desire to eat sweet food in youth (42). The authors hypothesized that sleep restriction increases carbohydrate intake *via* an increased hedonic value of these foods (42). Furthermore, Zuraikat et al. (20) postulated that changes in taste sensitivity could contribute to the impact of sleep restriction on food intake. However, more evidence is needed to support this hypothesis.

Regarding sleep patterns, we observed that later sleep/wake schedules were significant predictors of poor diet quality. The latter would be in agreement with other studies that highlight the role of late bed and wakeup times as predictors of poor diet quality (15, 43, 44). Particularly, we found that participants with a later sleep pattern were more prone to skip breakfast, which is not surprising since late diurnal preference has been related to a shift in food intake toward later times of the day and higher odds of breakfast skipping (15, 43, 45). Although it is plausible that children who go to bed late prefer to trade breakfast for extra sleep time (15), this behavior should not be encouraged, considering that breakfast skipping is significantly associated with obesity and poor sleep quality (15, 45, 46). This is also consistent with our observation regarding the increase in SDSC scores among children who skipped breakfast. Although circadian misalignment is a plausible mechanism linking breakfast skipping to poor sleep outcomes, the mechanisms have yet to be elucidated (46).

Finally, our findings regarding the association between sleep disorders and increased BMI are in line with the literature (9, 16, 47). Note that sleep disturbances increase morning cortisol levels and reduce both insulin sensitivity and growth hormone secretion, supporting the association between poor sleep quality and obesity (40). Moreover, a recent review on factors affecting sleep quality in children highlighted that sleep-related breathing disorders, such as obstructive sleep apnea syndrome or sleep hypoventilation syndrome, can increase sleep latency and sleep fragmentation and shorten sleep duration (48). The latter would be in line with our observation of the associations between increased BMI with greater disorders of initiating and maintaining sleep and sleep-related breathing disorders.

Our study has certain limitations to consider when interpreting our findings. First, its cross-sectional nature limits the ability to establish causality. Second, we acknowledge that convenience sampling is a limitation of the generalizability of our results. It has been pointed out that respondents usually have a higher educational level and socioeconomic status, compared to non-respondents (49). Along these lines, another limitation is the fact that a potential confounding factor, such as socioeconomic status, was not evaluated. Furthermore, BMI, sleep disturbances, and eating behaviors, were assessed through questionnaires. Future studies should consider a more objective measurement method, including body composition and actigraphy. The latter is important considering that while the parental description of a child's sleep appears to be adequate with regard to symptoms, self- or parent-reported data are moderately correlated with actigraphy (50–52). Our strengths lie in the sample size, which was large enough to provide sufficient strength for the associations of sleep disturbances with BMI, eating behaviors, and diet quality. Additionally, it is important to note the homogeneity regarding gender distribution (50% girls), which may be representative of the child population.

In summary, our results indicate that when it comes to sleep and eating habits, all dimensions matter. Here, we showed that late sleep patterns, short sleep duration, and higher sleep disturbances were significantly related with poor diet quality. Furthermore, we showed that sleep disturbances play an important role in eating behaviors. As such, greater responsiveness to satiety and slowness in eating were related to more sleep disturbances. Additionally, we observed that the greater the sleep disturbances, the greater the responsiveness to food. Note that this association that was found to mediate the relationship between sleep and obesity in school-aged children. The latter suggests that children with higher sleep disturbances were more likely to eat palatable foods or respond to food cues, which in turn would lead to weight gain. However, there are still many questions regarding the mechanisms and potential confounders in the sleep-obesity relationship, and reverse causality cannot be excluded either. Therefore, these findings could open a new framework for future nutritional intervention studies in pediatric population, which could focus on sleep hygiene as a strategy to improve children's eating habits, as well as their BMI.

## Data availability statement

The raw data supporting the conclusions of this article will be made available by the authors upon request to interested researchers.

## Ethics statement

The studies involving human participants were reviewed and approved by the Ethics Committee of the University of Barcelona (IRB00003099). Written informed consent to participate in this study was provided by the participants' legal guardian/next of kin.

## Author contributions

Conceptualization and methodology: MI-P and MZ-R. Data acquisition: CR-C and AS-O. Investigation, formal analysis, data curation, and writing—original draft preparation: CR-C and MZ-R. Writing—review and editing, supervision, project administration, and funding acquisition MI-P. All authors have read and agreed to the published version of the manuscript.

## Funding

CR-C was supported by the National Agency for Research and Development (ANID)/Scholarship Program/DOCTORADO BECAS CHILE/2019—72200134. The project was financed by Laboratorios Ordesa S.L. (FBG311143). However, Laboratorios Ordesa had no role in the design of the study, nor in the analysis or interpretation of the data.

## Conflict of interest

The authors declare that the research was conducted in the absence of any commercial or financial relationships that could be construed as a potential conflict of interest.

## Publisher's note

All claims expressed in this article are solely those of the authors and do not necessarily represent those of their affiliated organizations, or those of the publisher, the editors and the reviewers. Any product that may be evaluated in this article, or claim that may be made by its manufacturer, is not guaranteed or endorsed by the publisher.
