# Supplementary material for: Sleep dimensions are associated with obesity, poor diet quality and eating behaviors in school-aged children
Source: Front Nutr. 2022 Sep 23;9:959503. doi: 10.3389/fnut.2022.959503 (PMC9539562; doi:10.3389/fnut.2022.959503)
Supplement: Supplementary file 1 [file Data_Sheet_1.pdf]

Figure S1. Path diagram for the total effect of sleep disturbances on the body mass index (BMI) and the indirect effects of sleep disturbances on the BMI through the potential mediation of food responsiveness, satiety response and slowness in eating. In the top diagram "c" is the total effect of exposure (X) on outcome (Y) ignoring the mediator (M). In the bottom diagrams, the mediation effect of sleep disturbances on the BMI through eating behaviors is shown, where "a" is the effect of exposure on mediator and "b" is the effect of mediator on outcome. Effect c' is the direct effect of exposure on outcome while adjusting for the mediator.

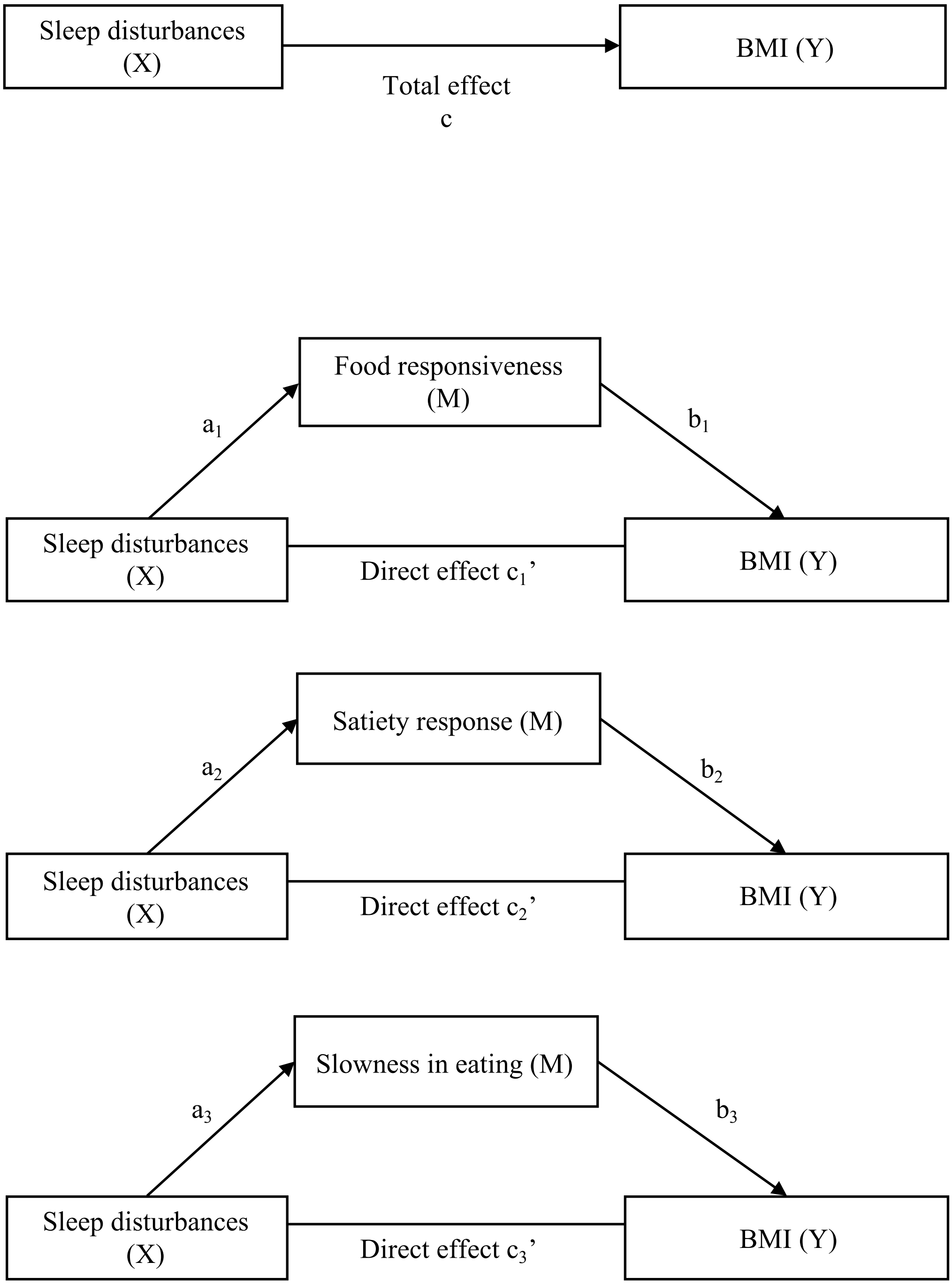

Table S1. Questions included in the shortened version of the Children's Eating Behavior Questionnaire presented according to the subscale to which they belong (28).

Subscale	Questions
Food responsiveness	Your child is always asking for food
	If allowed to, your child would eat too much
	Given the choice, your child would eat most of the time
	Even if your child is full up, he/she finds room to eat his/her favorite food
	If given the chance, your child would always have food in his/her mouth
Satiety responsiveness	Your child has a big appetite
	Your child leaves food on his/her plate at the end of a meal
	Your child gets full before his/her meal is finished
	Your child gets full up easily
	Your child cannot eat a meal if he/she has had a snack just before
Slowness in eating	Your child finishes his/her meal quickly
	Your child eats slowly
	Your child takes more than 30 minutes to finish a meal
	Your child eats more and more slowly during the course of a meal

Table S2. Definition of sleep factors derived from the Sleep Disturbance Scale for Children.

Factor	Definition
Disorders of initiating and maintaining sleep	Describes sleep duration, and latency, as well as any problems related to bedtime resistance, anxiety to go to bed, and problems related to night awakenings. This factor was evaluated through 7 items.
Sleep breathing disorder	Describes any breathing problems during sleep, including sleep apnea, and/or snoring. This factor was evaluated through 3 items.
Arousal disorders	Describes problems with nocturnal awakenings related to parasomnias, such as sleepwalking, sleep terrors, and/or nightmares. This factor was evaluated through 3 items.
Sleep-wake transition disorders	Describes any problems during sleep related to parasomnias, including hypnagogic hallucinations, vocalizations, and/or complex motor behaviors. This factor was evaluated through 6 items.
Disorders of excessive somnolence	Describes problems related to difficulty in waking up, tired when waking up, sleep paralysis, daytime somnolence, and/or sleep attacks. This factor was evaluated through 5 items.
Sleep hyperhidrosis	Describes problems related to sweating, such as falling asleep sweating, and/or night sweating. This factor was evaluated through 2 items.

Table S3. Correlations between the body mass index and factors of Sleep Disturbance Scale for Children in school-aged children.

	BMI, kg/m^2	
	**r**	**P-value**
Disorders of initiating and maintaining sleep, score	0.102	**0.024**
Sleep breathing disorders, score	0.096	**0.032**
Arousal disorders, score	0.041	0.333
Sleep-Wake transition disorders, score	0.059	0.186
Disorders of excessive somnolence, score	0.046	0.287
Sleep hyperhidrosis, score	0.084	0.059

BMI, Body mass index. The table shows Pearson correlation coefficient (r). Statistical analyses: partial correlations controlled for age, gender, and physical activity. Significant p-values are shown in bold.
